# Implementing public involvement throughout the research process—Experience and learning from the GPs in EDs study

**DOI:** 10.1111/hex.13566

**Published:** 2022-07-27

**Authors:** Bridie Angela Evans, Andrew Carson‐Stevens, Alison Cooper, Freya Davies, Michelle Edwards, Barbara Harrington, Julie Hepburn, Tom Hughes, Delyth Price, Niroshan A. Siriwardena, Helen Snooks, Adrian Edwards

**Affiliations:** ^1^ Swansea University Medical School Swansea University Swansea UK; ^2^ Swansea University Medical School, PRIME Centre Wales Swansea University Swansea UK; ^3^ Division of Population Medicine, School of Medicine, PRIME Centre Wales Cardiff University Cardiff UK; ^4^ Public Contributor, c/o Swansea University Medical School Swansea University Swansea UK; ^5^ John Radcliffe Hospital Oxford UK; ^6^ School of Health and Social Care University of Lincoln Lincoln UK

**Keywords:** consumer involvement, evaluation, public and patient involvement, research

## Abstract

**Background:**

Public involvement in health services research is encouraged. Descriptions of public involvement across the whole research cycle of a major study are uncommon and its effects on research conduct are poorly understood.

**Aim:**

This study aimed to describe how we implemented public involvement, reflect on process and effects in a large‐scale multi‐site research study and present learning for future involvement practice.

**Method:**

We recorded public involvement roles and activities throughout the study and compared these to our original public involvement plan included in our project proposal. We held a group interview with study co‐applicants to explore their experiences, transcribed the recorded discussion and conducted thematic analysis. We synthesized the findings to develop recommendations for future practice.

**Results:**

Public contributors' activities went beyond strategic study planning and management to include active involvement in data collection, analysis and dissemination. They attended management, scrutiny, planning and task meetings. They also facilitated public involvement through annual planning and review sessions, conducted a Public Involvement audit and coordinated public and patient input to stakeholder discussions at key study stages. Group interview respondents said that involvement exceeded their expectations. They identified effects such as changes to patient recruitment, terminology clarification and extra dissemination activities. They identified factors enabling effective involvement including team and leader commitment, named support contact, building relationships and demonstrating equality and public contributors being confident to challenge and flexible to meet researchers' timescales and work patterns. There were challenges matching resources to roles and questions about the risk of over‐professionalizing public contributors.

**Conclusion:**

We extended our planned approach to public involvement and identified benefits to the research process that were both specific and general. We identified good practice to support effective public involvement in health services research that study teams should consider in planning and undertaking research.

**Public Contribution:**

This paper was co‐conceived, co‐planned and co‐authored by public contributors to contribute research evidence, based on their experiences of active involvement in the design, implementation and dissemination of a major health services research study.

## 1 BACKGROUND

Public involvement across the research cycle is encouraged to improve the relevance, quality and accountability of health and care research.^1^, ^2^, ^3^ Many research funders expect evidence of involvement in developing study proposals and in planning how involvement will occur throughout a funded study.^2^, ^4^, ^5^ However, there continue to be barriers to developing and undertaking impactful research with patients and public members.^6^, ^7^, ^8^, ^9^

Conceptual models of public involvement in research highlight the different levels of involvement from none to full control of research.^10^, ^11^ Involvement and partnership require power to be shared within research teams.^12^ Some public contributors report negotiating their influence by using their experience, leading to queries of professionalization.^13^ Others argue that communication skills sensitize research teams to patient experiences, a role that goes beyond simply presenting patients' encounters with health care services.^14^

To address concerns about the quality and effectiveness of public involvement processes, several guidelines, standards and value statements have been published, most prominent of which is the UK Standards for Public Involvement.^15^, ^16^, ^17^ Researchers and policy experts also recommend that public involvement in research is thoroughly assessed, to build an evidence base and inform future practice.^15^, ^18^ The GRIPP 2 reporting checklist is one tool for ensuring that public involvement is consistently described in published papers requiring, as a minimum, a summary of public involvement activity.^19^ The checklist does not consider the quality or effects of reported involvement. Detailed accounts of how public involvement is undertaken within research studies remain limited^20^ and usually focus on specific involvement roles.^18^, ^21^, ^22^, ^23^, ^24^ Descriptions of public involvement across the whole research cycle of a major research study are uncommon.^25^ More evidence about the context, process and effects of public involvement in research is needed.^21^, ^26^ There are divergent views over the value of, and approaches to, assessing effects of public involvement,^27^, ^28^, ^29^ including defining core features, activities and mechanisms^30^, ^31^ and encouraging feedback and mutual learning.^32^, ^33^

We undertook a large research study (realist evaluation) involving three phases of data collection and analysis over 4 years. Our study evaluated models of care in which general practitioners work in or alongside Emergency Departments at UK hospitals.^34^ Our successful application to the National Institute of Health Research (NIHR) Health Service and Delivery Research (HS&DR) programme (project 15/145/04) included a description of proposed public involvement in delivering the study. Our objectives for public involvement were to ensure that our research remained relevant to health service users by including public and patient experiences and perspectives throughout research planning, implementation and dissemination. We also determined that our research should be acceptable, understandable and feasible, to be of the highest quality. These objectives informed our plan for public involvement. This plan was developed with two public co‐applicants who also had extensive input throughout planning the research design.

In this paper, we describe how we involved public contributors in our study. We consider how this involvement evolved from the way we envisaged at the study outset, the experiences of all co‐applicants collaborating in the research and present lessons for future practice.^19^ This first‐hand account of public involvement^22^, ^24^, ^26^ is co‐authored by public and academic co‐applicants and conveys multiple perspectives from our collaborative work on this study. Two authors are public contributors (B. H., J. H.); 10 authors are academics, clinicians, policy makers and health service managers. The author group has worked together for 6 years.

## 2 AIM

This study aimed to describe:

1. how we implemented public involvement within our research study;
2. reflections on the process and effects of public involvement in a large‐scale multi‐site research study; and
3. learning for future involvement practice.

## 3 METHOD

### 3.1 Terminology

We use ‘public contributor’ to describe the roles of J. H. and B. H., who were co‐applicants in the general practitioner (GP)s in EDs research study and the two other individuals who joined the independent Study Steering Committee. We occasionally distinguish J. H. and B. H. as public contributor co‐applicants to avoid confusion. We use ‘public and patient members’ to describe the individuals who attended stakeholder events and contributed to those stages of the research.

### 3.2 Initial public involvement approach

Before planning our research, we recruited two public contributors (J. H. and B. H.), with experience of using primary and secondary health services, from the Public Involvement Community supported by Health and Care Research Wales (HCRW).^35^ Our involvement plan was developed alongside research design and preparation of the funding application. J. H. and B. H. joined the research development group. They took part in all discussions about the proposed research. They shared responsibility for authoring plans for public involvement in the study, including reviewing the proposed budget. They drafted the plain English summary. They were named research co‐applicants on the outline and full applications.

In our study, we proposed the following approach to public involvement. We committed to continuing our relationship with the two public contributors we named as co‐applicants on the study. We intended that they would maintain an equal status among all co‐applicants within the research management team with full opportunity to contribute to strategic and operational decisions throughout the research. We also proposed they would join subgroups to plan data collection, review early findings, co‐plan stakeholder events and lead the public strand of our dissemination activities. At their suggestion, we expected them to host pre‐meetings to brief and support public and patient members attending our two stakeholder events.

In addition, we planned to recruit two different individuals through the HCRW Public Involvement Community^35^ to join our Steering Committee as public contributors and providing independent oversight of our study. Finally, we intended to recruit seven public and patient members to attend two stakeholder events alongside equal numbers of other stakeholder groups (Table 1).

**Table 1:** Planned and actual public involvement in our study

Involvement plan	Change	Variation or addition
*Study management and delivery*		
2 public contributors at the research management group—strategic and operational responsibility over study	No change	Undertaken in line with plan
2 public contributors at four subgroups: data collection; review findings; plan stakeholder events; and dissemination	Change	Six subgroups convened and included public members. Additional groups: manage rapid realist review; review interview data
Support 7 patient and public members at two stakeholder^a^ events	Change	Additional roles undertaken by public contributors: recruiting 15 public members; co‐planning the agenda and room layout to address public needs; facilitating discussion groups; and co‐presenting
Dissemination—2 public contributors lead public strand	Change	Dissemination activities extended across all aspects of the study and included:
		• Oral and written presentations of public involvement in study • Co‐authors of research papers • Comments on reports to funders • Presentations to stakeholders^a^ • Inputting patient perspective to dissemination strategy • Preparing lay summaries of all research outputs
*Study oversight and advice*		
2 public contributors at the Study Steering Committee	No change	Undertaken in line with plan
*Additional public input*		
7 public members (excluding study public contributors) at two stakeholder^a^ events	Change	15 public members attended the 2‐day‐long stakeholder* events; 6 attended the first; 10 attended the second; and 1 of these people attended both. These individuals shared patient stories to explain patient priorities and decision‐making processes when seeking emergency health care
*Public involvement processes and effects*		
Named academic lead for public involvement to support public members	No change	Undertaken in line with plan
	New	Public Involvement Team meetings to plan, review and operationalize public involvement throughout the study (2 public contributors and 1 public involvement lead)
	New	2 public contributors conducted audit of public involvement in the study and amended processes in light of results
	New	2 public contributors collected data on processes and effects of public involvement in the study and reported these
*Total planned number of public individuals involved*		*Total actual number of public individuals involved*
11		19

a Stakeholders at these events included public and patient members, health service managers, clinicians, policy makers and researchers.

We calculated a budget (0.5% of total research bid) to provide honoraria and reimburse all expenses. We named an academic co‐applicant (B. A. E.) in the funded post of Public Involvement Lead. Her role included liaison with public contributors, ensuring accessible opportunities for their involvement and offering training and support in the event of distress or difficulty. We committed to standards of best practice, such as providing honoraria, accessible information and a named contact person (see Supporting Information: Appendix 5 for further examples).^15^, ^36^

### 3.3 Data collection

To understand how we implemented or diverged from our initial public involvement plan, team members made comprehensive notes throughout our study. We documented public involvement activities in our meeting minutes and any reported effects on study processes. We made a timeline of public involvement activities in our study by compiling a table recording the activity and effects.

To understand experiences and effect of public involvement in the study, we held a group interview (Supporting Information: Appendix 1) with co‐applicants attending a regular (online) study management meeting during the last 6 months of the funded study (9/14 participated: 6 university staff; 3 clinical/policy experts). Questions were developed and administered by B. H., J. H. and B. A. E. based on a public involvement effects framework.^37^ B. H. and J. H. responded to questions and comments at the end of the interview. With participants' consent, the discussion was audio‐recorded and transcribed using a facility within Zoom software. We encouraged interview respondents to be frank in their answers and invited them to send comments by email to a third party if they felt uncomfortable at any point.

### 3.4 Analysis

J. H., B. H. and B. A. E. reviewed records describing the roles and outcomes of public involvement, compared to the original involvement proposal. We undertook thematic analysis of the group discussion informed by the public involvement effects framework.^37^, ^38^ B. A. E., J. H. and B. H. read the transcript, coding text with the underpinning framework and adding any new codes. Through discussion, we generated themes and interpretation, taking a critical stance to test and confirm findings.^39^, ^40^, ^41^

We then brought together all data about implementation processes, experiences and effects to consider what we had learnt about involvement in our study. We identified what contributed to effective involvement and prepared recommendations for implementing public involvement, based on our findings.

### 3.5 Reporting

We present our account of public involvement in three parts: (1) the organizational structure through which we implemented involvement; (2) involvement processes and their effects on conduct of the study; and (3) reflections by team members on the experience of public involvement and how they felt it affected the study.

We selected quotations from our group interview to be illustrative and typical of respondents' comments unless otherwise stated. Quotations are identified by numbers 01–09.

## 4 RESULTS

### 4.1 Implementing involvement

1. Public involvement throughout this study was more extensive and detailed than envisaged.
2. Numbers of public and patient individuals involved increased from 11 (planned) to 19 (actual).

We convened five forums and ensured public involvement at all stages (Table 1).

1. Research management group: all co‐applicants (including two public contributors) plus research staff met every 1–2 months, taking all strategic and implementation decisions. This was in our original plan.
2. Study Steering Committee: two independent public contributors plus methodological, clinical and policy specialists met every 6 months, providing oversight and advice to the research team. This was in our original plan.
3. We had planned for public involvement in four subgroups. In the study, six subgroups were convened. New tasks included undertaking the rapid realist review and reviewing interview data.
4. Stakeholder workshops—proposed role, to support public members at the events, was extended. Additional roles included recruiting public members, co‐planning the agenda and room layout to address public needs, facilitating discussion groups and co‐presenting.
5. We convened annual Public Involvement Team meetings. This regular forum was not originally planned. The Public Involvement lead coordinated these meetings and attended with the two public contributor co‐applicants. At the meetings, the team planned and reviewed the role and operation of public involvement in the study, identified and accessed support and training, including any issues causing distress or difficulty for individuals, and identified opportunities to extend public involvement in the study. Briefing sessions were identified and provided, but no formal training was requested by public contributors. Members co‐authored a Role Description document, which they updated annually and reported to the research management group (Supporting Information: Appendix 2).

### 4.2 Processes of involvement

1. Joint working at all stages of delivering the research enabled public views to be included in discussions and decisions, from preparing the ethics application through to dissemination.
2. Public contributors extended the opportunities for involvement in this study, enabling public and patient views to be more fully included.

#### 4.2.1 Study management, delivery, dissemination and scrutiny

##### Role

**Figure 1:**
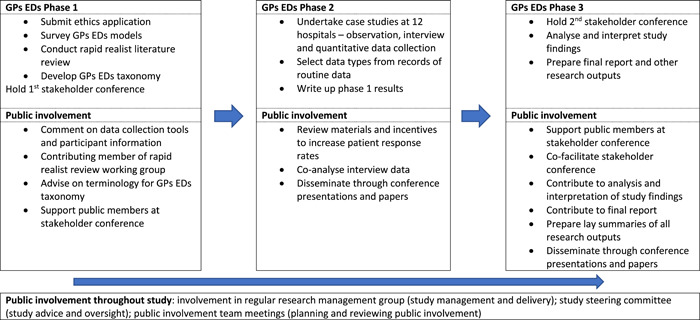
Public involvement in the research phases of the GPs EDs study. GP, general practitioner

Public contributors were involved at all stages of the research cycle, from developing the research proposal to reporting and dissemination (Table 1). Public involvement in the three phases of the study is shown in Figure 1. Public contributors' activities included:

1. reviewing all participant information and data collection tools and processes that were submitted for Research Ethics Committee approval,
2. planning and participating in activities within each study phase: the rapid review of evidence, a survey of hospitals, selection of case study sites, case study data collection and analysis and overall management and scrutiny,^42^, ^43^, ^44^, ^45^, ^46^
3. planning and delivering the two stakeholder events, including developing materials for workshops during each day, facilitating some of the round‐table discussions and presenting the workshop sessions alongside researchers,^47^
4. dissemination activities including reports to the study funders, producing a range of accessible written and oral outputs during the study, preparing lay summaries of all academic papers [see GPs in emergency department (ED)s (primecentre.wales)], co‐authoring publications^45^, ^46^, ^48^ and planning for dissemination at study end and
5. scrutiny through the Study Steering Committee.

##### Effects on delivering the research

In many instances, the contributions made by public contributors were in team meetings where their views were sought and heard, playing a part in debate and collective decision‐making. In these situations, it was not generally possible to determine whose opinion or contribution caused, or was the consequence of, which decision. In other instances, we could describe the role and outcomes of public involvement in the study. In Table 2, we summarize public involvement at all stages of the study and any effects of public contributions by J. H. and B. H. In Supporting Information: Appendix 3 (Boxes 3.1–3.4), we provide a detailed account of four of those involvement episodes.

**Table 2:** A summary of the input and effects following public involvement in study management, implementation and scrutiny

Activity summary	Public contributor input	Effects
Preparation of ethics application	Co‐drafted and reviewed patient‐facing materials	Amended wording to participant information material and data collection tools changed the detail of information provided and ease of reading
Clinical directors survey^39^	J. H. member of working group reviewing the structure of the questionnaire by e‐mail	Amendments to the questionnaire and accompanying information
Rapid review^37^	B. H. member of working group, took part in telephone meetings	Patient focus included in discussions to generate ‘initial rough theories’
Selection of marker condition (inclusion criteria for analysis)^41^	Identified need for additional marker condition, to be used in analysis of patient outcomes	Suggested using feedback from Stakeholder Event to identify a condition with resonance to clinical, managerial and patient attendees. This resulted in ‘headache’ being identified and used
Monitoring response rates to patient interviews^38^	Assessed opportunities to encourage response rates because of low patient numbers	Patient information sheets and recruitment letters reworded
		Financial incentive offered to increase patient recruitment
		Letters to be sent on hospital letterhead in white envelopes instead of being university‐badged
Qualitative analysis^38^	Involved in identifying themes and synthesizing data	Identified limitation that patient respondents were likely not to reflect all ED attendees since only patients perceiving their behaviour was positive would consent to interview
		Patient interview results will be reported across the study
		Highlighted complexity of models of general practitioners in EDs and local variations
		Confirmation of research themes in line with that of the researchers: quality‐check of analysis process
		Gave researchers insight into data quality, patient experience and complexity of the models reported to help J. H. and B. H. in their role
		Researchers identified additional checking role during the theory‐building stage of analysis and interpretation
Dissemination	J. H. facilitated collection of patient views to inform dissemination planning	J. H. presented to the SUPER public/patient group^43^ to explore patient views on how and when to disseminate study results. The following feedback was incorporated within the dissemination strategy:
		• Wait until big findings; interim results less meaningful to patients • Make friends with media to maximize dissemination opportunities at the end of the study
	Prepared lay summaries of all academic outputs	Accessible information about study findings throughout the study, uploaded to a project webpage and widely publicized (http://www.primecentre.wales/gps-in-eds.php)
	Input into Dissemination, Publication and Engagement Strategy	Dissemination and Publication strategy widened to include engagement. Equal opportunity to co‐author outputs confirmed. Co‐authored conference presentations and papers. Contacted the Communications section of Public Involvement and Engagement, Health and Care Research Wales. Volunteered to facilitate engagement with media

Abbreviation: ED, emergency department.

#### 4.2.2 Developing public involvement in the study

##### Role

Public contributors played an active role in the process of planning and implementing public involvement throughout the study. They decided how the involvement processes, summarized in the research application, were undertaken in practice. They held discussions at Public Involvement team meetings and undertook an annual Public Involvement review that initiated additional activities to extend the role of public contributors and strengthen the practice.

##### Effects on processes of public involvement

Public involvement in the study was more extensive than proposed in the research proposal and funding application. The Funder described it as ‘a fine example of embedding public and patient involvement (PPI) throughout the course of the research’ after receiving routine study reports. Activities that were extra to those proposed in the original study proposal included:

1. planning and managing PPI input through annual review meetings and regular contact between the PPI coordinator and public contributors,
2. collecting data on effects of public involvement during the study,
3. conducting an audit of public involvement, against the UK standards for public involvement and^15^
4. monthly research updates from the core team, at the request of public contributors, to keep wider team members abreast of study progress.

In Table 3, we summarize instances where public contributors amended processes of involvement in the study. In Supporting Information: Appendix 4 (boxes 4.1–4.4), we detail four case studies.

**Table 3:** A summary of the input and effects of public contributors in developing public involvement in the study

Activity summary	Public Contributor input	Effects
Planning PPI input^a^	Actively involved in implementing and reviewing how public members were involved in the study	Held annual PPI team meeting
		Reviewed and amended PPI role; recorded changes in an updated role document
		Confirmed role was co‐produced in response to study requirements and opportunities.
		Extended role to include analysis, interpretation, synthesis and dissemination of results, co‐producing and defining role, undertaking PPI Standards audit, Instigating annual training review
Recording effects of PPI^a^	Developed way to record data about PPI effects	Research team adopted a regular research team agenda item—‘PPI impact and effects’—with content noted in ‘Impact Box’ and reported in meeting minutes. Researchers and PPI team to contribute to evidence of effects
Stakeholder event phase 1	Oversaw recruitment and participation of public members at event	Devised recruitment strategy—developed recruitment information, who to target, follow‐up/thank you contacts, ensured financial support offered
		Amended pre‐information to public participants to ensure that information was informative and easy to understand
		Co‐facilitated discussions by public participants
Stakeholder event phase 2	As for stakeholder event phase 1 (above) plus clarified purpose of the second event and expected contribution of patient attendees.	Involved in meetings and email discussion querying the purpose of the event and need to avoid tokenistic involvement from patient attendees. Confirmed scope of event and commitment from CI to meaningful involvement. Resulting changes included:
		• amended recruitment letter and timetable for responses, • amended workshop format with greater mixing of PPI attendees with other stakeholders and at least 1 PPI contributor on each table and • J. H. and B. H. to co‐present and co‐facilitate the meeting
Public Involvement standards audit^a^	J. H. led audit of public involvement and recommended changes in line with national standards. She reviewed actions after 12 months and reported back to the research team (Supporting Information: Appendix 5)	Following the audit, the team:
		• recruited more diverse public participants at stakeholder events • improved communication with a monthly study update • undertook PPI training reviews and • involved public contributors in producing plain English summaries of all research outputs to improve study dissemination
Within‐study communication^a^	Noted from PPI audit the difficulty of remaining well informed of study activity between quarterly meetings	Monthly research updates were provided, available to all team members
Study scrutiny	2 public contributors on the Study Steering Committee (SSC)	Effects of SSC public involvement was recorded and reported

Abbreviation: PPI, public and patient involvement.

^a^ New activity.

### 4.3 Research team members' experiences of involvement

1. Research co‐applicants said that public involvement had exceeded their expectations and reported positive feelings including pride and self‐worth arising from the experience.
2. Public involvement enabled changes to study processes, such as patient recruitment and dissemination, but also presented challenges when expectations exceeded resources.
3. Respondents identified factors they considered to have facilitated involvement in the study.

Nine people participated: six university staff (chief investigator, researchers, administrator) and three clinical/policy experts. The two public contributors addressed discussion points at the end.

#### 4.3.1 Expectations and experience

All respondents reflected that public involvement had outgrown their early expectations, with more opportunities created to include public contributors than first proposed. Compared to other research projects, they found these public contributors to be more actively involved in study management and delivery.

even when our expectations have risen (with) changing general expectations about what is effective patient and public involvement and with the national standards…there has been more than expected contribution. (05)

They believed that this resulted in strong team working, with public contributors collaborating equally alongside other stakeholders in discussions and decision‐making throughout the study.

seamless, it didn't feel like something separate…part of the study. (06)

I was impressed at how much involvement there was in all the different stages of the project. So, it certainly wasn't just like in a tick box. (09)

One respondent gave a different view, uncertain whether public members fulfilled all expectations and roles because they had not been challenging enough and believed they were in danger of becoming too integrated within the study team.

Are you becoming professional researchers? Or are you still genuinely spokespeople for public and patients… that's your job isn't it? (08)

#### 4.3.2 Effects—What difference did it make?

Respondents identified a number of roles and activities affecting the research study. These included supporting patient recruitment, reviewing qualitative data, planning and co‐delivering stakeholder conferences and clarifying language and messages in dissemination.

to sense check what we were doing, to challenge us about our methods, to think about how we were going to recruit patients, really great feedback (on what) we presented in our initial findings, helping with stakeholder events. We couldn't have done that without the PPI team, to have had such good recruitment and to have got such a diverse group to attend for stakeholder events. (03)

Respondents recalled several instances where public input changed a discussion or decision. One recalled how a public contributor had ‘rescued us’ (07) during complicated discussions about terminology, to suggest a simpler descriptive phrase, which was then used in all outputs. Another respondent altered data presentation after a public contributor said that the findings were not easily understood. Some respondents felt that public contributors in the team shifted researchers' awareness to focus on patient experience alongside statistical hospital data.

4.4

Participants also reported changes to their view of the research.

to have PPI there is a constant and useful reminding of the wider context in which this study sits, that's always helpful…it's not just a statistical project with the few bits added on, it's a much wider thing, much more important thing. (02)

Several remembered instances where presence and input from public members reminded team members that the research was for patients' benefits. One respondent said that he now advocated for public involvement in other studies he was working on because he valued this effect on team outlook.

you've kept the focus on what really matters, actually what's relevant to this project, and it's better service delivery, you kept us on track with that… it started me questioning it (PPI) in other areas. (07)

While some effects appeared intangible, respondents suggested that these were interlinked with concrete changes and that effects were revealed in a chain of small events, which led to things being done differently. This respondent recalled team discussion about low recruitment numbers and that ideas from multiple perspectives convinced the lead researchers to try a different approach.

To me, one of the most concrete ones was the issue about recruitment of patients in the ED for interviews, and different strategies, including the incentives. That was generated from the PPI contributors, actually including the Study Steering Committee PPI members. But then I think it was strongly supported. The initiative to bring that in was led by the PPI contributors, and gave us the confidence to then put that to the ethics committee. (05)

#### 4.4.1 Motivation and fulfilment

Respondents' comments during the interview suggested feelings of pride for the effective way in which public contributors had been involved in the research, in contrast to their other experiences. They spoke warmly of how meetings had felt cooperative: the sense of shared endeavour was strengthened because everyone was contributing in different but equally valuable ways. That public involvement exceeded expectations—‘extra value' (07)—was considered a benefit, respondents reported.

they contributed as equal members in that team… more than I was expecting but that was a good thing. (03)

They celebrated how public involvement was integrated within study delivery.

it's just not one thing, it's not just PPI involvement, it is of multi‐dimensional contribution. (08)

Public contributors acknowledged how their sense of worth within the study team increased through involvement. This also nurtured their motivation and enthusiasm, further building the team bond and sense of shared purpose. Their sense of self‐worth was also enhanced by the respect given to their contributions and the atmosphere of mutual respect within the team.

you've demonstrated that you have been open to us, developing the role. I think we have felt listened to and respected. (PPI 02)

we were able to make suggestions, and they were welcomed and incorporated, where possible, and we were given real and not token roles. (PPI 01)

#### 4.4.2 What made it work?

Respondents identified factors that enabled perceived effective public involvement in the study:

1. Commitment across the co‐applicant team to public involvement, led by the Chief Investigator.
2. Having a university staff member to lead public involvement and to support individuals in their role.
3. Providing public contributors with support and training in the skills required to undertake the role.
4. Including public contributors as co‐applicants, equal at meetings and visible alongside other team members, which made them integral to conducting and delivering the research.
5. Having regular face‐to‐face meetings (and online from 2020), building up a relationship within the team so that people felt they could work together.
6. Public contributors being able to work to researcher deadlines, flexible and prompt in responding to requests for input.
7. Public contributors being confident enough to articulate their views including challenging other team members.

#### 4.4.3 Challenges

Managing study resources within budget and timescales while also meeting rising interest in and expectations of public involvement was a challenge for researchers. Tight deadlines, staff availability and fixed costs meant that research staff struggled to work with public contributors as frequently as, or when, they wished.

expectations and resources, there weren't resources to provide the level of involvement for yourselves that we want, and the national standards advocate. (05)

One respondent questioned whether public contributors had been too closely aligned with the research team. The effective relationships and sustained involvement were ‘sucking you into our ways, in our language, and our enjoyment of research’ jeopardizing their role as ‘genuinely spokespeople for public and patients’ (08). Respondents also discussed the challenge of recruiting diverse public contributors.

Public contributors responded by describing the balancing act of maintaining an outside perspective but gaining the communication and research skills to effectively contribute to the collaboration.

finding the right place, that is not too involved, but that learns enough about a project to make informed contributions. (PPI 02)

Experience of illness and accessing care remained a defining part of their identify, ‘we've been to A&E, we've been to the GPs’ (PPI 02) but they also needed other skills to voice their contributions within research teams, they said.

the more of this sort of work you do, the better you get it actually challenging researchers, because it takes a lot of confidence to do that, when you start off you're not very confident. (PPI 01)

## 5 DISCUSSION

### 5.1 Summary of findings

In this study, we had a structured approach to involving public contributors that enabled them to have roles in project delivery and dissemination and in how public involvement was undertaken. Public contributors were actively involved throughout the study, taking on more research roles than was envisaged when the study was designed. This enabled changes to study processes, such as patient recruitment and dissemination. It made research co‐applicants more aware of patient views and outcomes when reporting results. They identified factors that facilitated this positive public involvement but also challenges when expectations exceeded resources.

### 5.2 Strengths and limitations

Data capture and analysis of public involvement in this study were led by the public contributors. This insider approach has been used by researchers and public contributors in other studies describing and reflecting on experience of public involvement in research^49^, ^50^, ^51^ and is encouraged for generating informed assessment of complex factors.^15^, ^16^, ^19^, ^52^, ^53^, ^54^, ^55^, ^56^ Nevertheless, we sought to minimize the risk of bias from potential conflict of interest, while capturing relevant data to address our study aim, in line with existing evidence. Our study records provided prospective data to capture real‐time information and minimize recall bias. We used a public involvement effects framework to structure data collection and analysis.^37^ We encouraged interview respondents to be frank in their answers and invited them to send comments by email to a third party if they felt uncomfortable expressing views verbally. One respondent did challenge the meeting consensus on some occasions and others did identify challenges. No emailed views were received.

### 5.3 Comparison with the existing literature

Best research practice is to involve public contributors in developing research from the earliest feasible stage and plan their involvement in the funded study.^57^ We believed that we had a robust and coherent plan that avoided tokenism.^58^ Co‐applicants believed that this expanded due to team dynamics, the supportive environment and the skills of those involved. Even though team members had mixed experiences and expectations of what public contributors' roles would be, there was consensus that it should be, and was, integral to the project. Having a structure and intention for involvement meant that we could respond to opportunities that deepened and widened the contributions that public members made. Commitment across the team and visible support from the Chief Investigator and senior staff underpinned our flexibility and opportunism. Support from the Public Involvement Lead boosted the involvement of public contributors. Despite the increase in public involvement, there continue to be few reports of such integrated involvement in decision‐making throughout a research study.^59^, ^60^

This placed more pressure on team resources to respond to additional involvement, despite having a well‐costed proposal including a PPI budget. Funders need to acknowledge the cost of genuine public involvement and accept that these may result in bigger research budgets.^15^, ^36^ Otherwise, research teams risk cutting other costs that are necessary to delivery of high‐quality and timely research, to appear competitive in the contested research application process, risking tokenistic involvement.

Extending public involvement in our study generated questions over whether these public contributors risked being too integrated within the research team and losing their outsider status. The notion that public contributors become too professional has been reported previously, but this view is not shared by the individuals who become involved in research.^61^, ^62^, ^63^ Researchers and user‐researchers report that experience differs from professionalism; the role requires skills that develop with practice and can be passed between contributors.^64^, ^65^, ^66^ That this interpretation persists may suggest that trust continues to be shaky among the research community and that individuals remain uncertain about the process and robustness of involvement. It may also reflect the practical challenges of delivering work to deadlines and with competing priorities, so that the requirement to involve public members and respond to their views becomes too demanding.^63^

The public contributors in our study said that they remained very conscious of their different skills and perspectives, including their health and care experiences.^67^ There is a long‐standing debate on who should be a public contributor.^68^ The current emphasis on including less‐heard voices overlaps these issues. People new to public involvement must gain enough knowledge and skills to make their voices heard. Power is recognized to rest with the research community and with it the responsibility to empower people and facilitate communication and interaction.^61^, ^63^ To overcome the competing demands on researchers, a Public Involvement Coordinator within a team can support patients and public members to be effective in their role.^15^ We included someone in this role within our team from the outset of project development. The role was considered by all partners to be critical to our positive experience, as this paper describes. Other barriers, which contribute to power imbalance and damage involvement processes and outcomes, were not identified by respondents in this study. These can include devaluing people, tokenism, controlling or minimizing information flows, all compromising clarity of role and partnership interaction.^18^, ^69^ Our prospective plan, subsequently extended by consensus and implemented with open discussion, provides an approach to building involvement through a research project.

Our account is a rare example of the story of public involvement in a research study that describes the actual activities that our contributors undertook.^26^, ^63^ With systematic record keeping and comprehensive meeting minutes, we were able to record specific changes because public contributors were involved in our study. We showed that they affected patient recruitment, interpretation and dissemination of findings. They also affected the context for discussions, providing new information or reassurance to support decision‐making and changing the awareness of research, clinical and policy study members so that the study gained a stronger focus on patient outcomes as it progressed.^70^ The interrelationship between tangible and intangible effects suggests the importance of integrating public members in a team so that they are part of ongoing discussions and decisions.^61^ The challenges of recording effects are well reported,^49^, ^50^ but can be overcome.

### 5.4 Implications for practice

Our study shows that there are some actions that support effective public involvement in health services research. Based on lessons from our experience, we make the following recommendations to support research teams to plan and undertake public involvement in large‐scale research studies. These recommendations may also be helpful for research teams when evaluating processes and effects of public involvement.

1. Ensure that public involvement is embedded from the earliest stage to allow influence in project design and implementation and to demonstrate equality within partnership from the outset.
2. Agree a team commitment to public involvement and document this through meeting documents and minutes.
3. Appoint a Public Involvement Coordinator, funded by the study budget, with the skills and responsibility to lead public involvement and support public individuals in their role.
4. Provide access to relevant and timely support and training so that public members have the skills and confidence to undertake their role.
5. Integrate public involvement in study delivery by including public members as equal members within research management structures and processes.
6. Make public involvement a standing item on meeting agendas to ensure that public contributors have an opportunity to raise issues above contributions made to other discussions.
7. Build a relationship within the research team to establish trust for collaborative working.
8. Clarify structures and ways of working so that public members and researchers have clear and realistic roles and expectations.
9. Review public involvement structures and processes during the study so that new opportunities can be identified and taken and additional support needs can be met.
10. Instigate simple data‐gathering of public involvement processes and outcomes and report these so that experiences can inform the emerging evidence base.

## 6 CONCLUSION

Public involvement in health services research is growing, but remains inconsistent and poorly reported. Using the skills, commitment and resources in our team, we extended our planned approach to public involvement in our research study. We identified benefits to the research process that were both specific and general. These led to changes in patient recruitment, data interpretation and dissemination, but also refocused the study to include more information on patient outcomes. Study co‐applicants reported positive experiences but also identified challenges, including matching rising expectations to available study resources. We identified good practice to support effective public involvement in health services research that study teams should consider in planning and undertaking research.

## AUTHOR CONTRIBUTIONS

This paper was conceived by Barbara Harrington and Julie Hepburn. Bridie Angela Evans led drafting of the manuscript, with detailed input from Barbara Harrington and Julie Hepburn. The manuscript received additional editorial input from Andrew Carson‐Stevens, Alison Cooper, Freya Davies, Michelle Edwards, Tom Hughes, Delyth Price, Niroshan A. Siriwardena, Helen Snooks and Adrian Edwards. All authors read and approved the final manuscript.

## CONFLICT OF INTEREST

The authors declare no conflict of interest.

### 1 ETHICS STATEMENT

Permission from a research ethics committee was not required, but the authors followed best practice in informing respondents, gaining consent and managing and reporting data.

## Supporting information

Supplementary information.

## Data Availability

The data that support the findings of this study are available from the corresponding author upon reasonable request.
