# Supplementary material for: Implementing public involvement throughout the research process—Experience and learning from the GPs in EDs study
Source: Health Expect. 2022 Jul 27;25(5):2471–84. doi: 10.1111/hex.13566 (PMC9615054; doi:10.1111/hex.13566)
Supplement: Supplementary file 1 — Supplementary information. [file HEX-25--s001.docx]

**Appendix 1: Group interview schedule**

**Public involvement in the GPs in EDs study**

**Topic guide for discussion with study co-applicants**

V3 incorporating items from the list produced by the Shared Learning Group on Involvement in Research in their document ‘Capturing the impact of involvement in research’ [37]. **The Shared Learning Group is made up of charities and aims to encourage information sharing on involvement (https://slginvolvement.org.uk/).**

Clarify that we are seeking

• positive and negative views

• views on the process of public involvement i.e. how public involvement happened

• views on the effects of public involvement on the study i.e. impact

• tangible and intangible effects of public involvement

NB: Confirm consent to record the discussion

Explain option for emailing comments via third person to encourage full range of views from respondents

**Aim of group interview: to gather views on the processes and effects of public involvement in the GPs in EDs study**

Q1: What has been your experience of public involvement during the GPs in EDs study?

What did you expect at the start?

How have your views changed over the course of this study?

What do you think has been the purpose of public involvement during the study?

Q2: In what ways has public involvement affected the study?

In what ways did you find PPI helpful?

In what ways did you find PPI difficult or disappointing?

What do you think it has it added to the study?

What has surprised you the most about public involvement in the GPs in EDs study?

- PROBE: Interested in effects on study delivery and on team collaboration
- Ask for EXAMPLES of what has been different because of public involvement

Q3: What has influenced the process of public involvement in the GPs in EDs study?

Leadership

Culture

Individuals

Roles

Other

- PROBE: Seek positive and negative influences

Q4: What lessons will you take forward to future projects?

Q5: Is there anything further you would like to add?

At end, BH and JH speak about their experience: felt able to go beyond their initial role – why do you think they could?

**Thank you for taking part.**

**Appendix 2: Role description document**


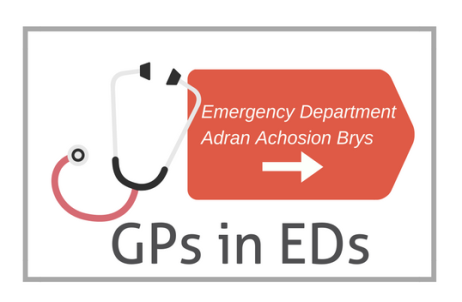


Role of public and patient contributors

in the GPs in EDs study

| **Version** | **Date** | **Authors** | **Circulated** | **Reasons for update** |
| --- | --- | --- | --- | --- |
| 1.0 | July 2017 | PPI team* | Study co-applicants | Annual PPI review (May 2018) |
| 2.0 | May 2018 | PPI team* | Study co-applicants | Annual PPI review (June 2019) |
| 3.0 | June 2019 | PPI team* | Study co-applicants | Annual PPI review  (April 2020) |
| 4.0 | April 2020 | PPI team* | Study co-applicants |  |

PPI team: Barbara Harrington, Public contributor

Julie Hepburn, Public contributor

Bridie Evans, PPI lead


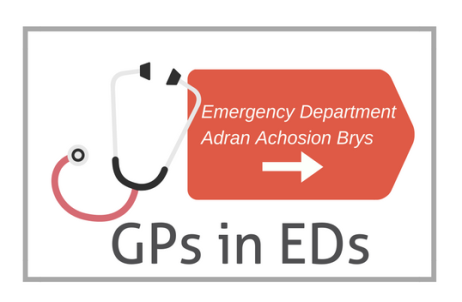


Role of public and patient contributors

in the GPs in EDs study

**Version 4: updated after annual review April 2020**

**Aim**

To ensure that: the **perspective** of patients and carers; the **relevance of the research** to patients and carers and **quality of patient care;** are recognised to be vital and fully considered throughout the study.

**Method**

Public and patient contributors will be actively involved throughout development and implementation of the study about GPs in EDs. The study team values their perspectives and contributions to complement the clinical, managerial and academic input of other contributors within the research partnership. Throughout their involvement, they will keep informed of study progress and prepare for meetings, by reading and reflecting on all documents circulated and asking questions where more information or clarity is needed.

They will also collaborate in the study in the following ways:

*Study development activities*

- Contribute to the development of the study and funding application and advise on public and patient involvement in undertaking the study
- Co-produce a lay summary of the proposed study for the funding application
- Support the funding application as co-applicants

*Study management*

- Attend and make an active contribution to regular Co-applicants Group meetings overseeing implementation of the research project
- Attend and make an active contribution to Co-applicant Working Group Meetings providing a patient/public perspective to guide decisions and recommendations, or take part in these processes by email when groups and meetings are not set up. These activities will relate to the main stages of the study and include: a Rapid Review Working Group; a Working Group to develop and review applications for ethical and governance approval and prepare the supporting documents; Analysis Working Groups; Dissemination Working Groups.
- Respond to requests by e-mail for comments and information between meetings
- Ensure that the interests of vulnerable patients and their carers are considered at all points in the study.

*Data collection*

- Review study documentation as required, in particular all information aimed at patients, to ensure it provides all the information the patient may need in a readily understandable way to enable them to make informed decisions about taking part in the study.
- Make suggestions that will help encourage successful recruitment and retention of patient participants
- Make suggestions that will improve the experience of participants being recruited to the study
- Review data collection plans to ensure issues relevant to patients and carers are captured. These will include plans for data collection from patients and data from other sources about the care and treatment of patients.
- Review structure and content of proposed questionnaires and interviews to ensure data of concern to patients are collected and to encourage participation and completed responses.

*Stakeholder events*

- Recruit and support patient and public delegates to attend and contribute to stakeholder meetings
- Be actively involved in the design of stakeholder conferences including clarifying role and purpose of PPI attendance and format on the day to enable their involvement
- Be actively involved the delivery of stakeholder conferences through facilitating and supporting delivery of parts of the programme
- Hold pre-meetings with patient and public delegates to assist their participation and enable them to make effective contributions
- Review design and delivery of stakeholder conferences from the perspective of PPI attendees

*Analysis, interpretation and presentation of results*

- Be involved in analysis of qualitative data to check validity of analysis from a patient and carer perspective
- With all study team members, be involved in synthesis, review and interpretation of study results and considering implications for patients and public members
- Contribute to the final study report and other study outputs and review draft documents

*Dissemination*

- Suggest channels through which the results of the study can be effectively communicated to patient groups and interested members of the public including considering media, message and content according to the target audience: for example, the lay press; Involving People newsletters; INVOLVE newsletters; National Association for Patient Participation bulletins; Welsh Ambulance Service Info Burst newsletters; voluntary organisations and charities.
- Take part in disseminating results of the study to enhance its reach and impact:
  - Disseminate about public involvement within the study (initial plan, changes during study with reasons and effects, overall learning)
  - Disseminate study findings to the public (in addition to academic, policy and service delivery audiences)
- Be involved in preparing information reporting progress and outcomes of the study including lay summaries and other articles and ensure, through reviewing these, that wherever possible, documents for the public and patients are written in plain English

*Study Scrutiny Committee*

- Two public members will join the independent Study Scrutiny Committee which will provide independent advise and oversight to the GPs in EDs study
- They will be actively involved as equal members of the Committee in all its activities.

*Co-producing, defining and evaluating the role of public and patient contributors*

The two public members who are study co-applicants will co-produce a document defining the role of public contributors in the GPs in EDs study. This document will be reviewed and updated annually and reported to other co-applicants. The project lead for Public and Patient Involvement will facilitate this process and the roles set out in the document. By reviewing and updating this document, the PPI team notes that public involvement in the study has evolved during the life of the study. This happened as opportunities for public contributors to be involved in research-related tasks were identified, which extended their role beyond that envisaged at study development. The support of co-applicants has been important in enabling this. The study lead for Public and Patient Involvement has facilitated these extra roles where necessary.

We will aim to follow best practice in our public involvement by auditing processes against the national Public Involvement Standards. This audit will be led by the public contributors, will involve key members of the research team and be reported, with request for comments and amendments, to all co-applicants.

The PPI team will describe and assess the role of public contributors in the study and report in the final report to the funders and in other outputs (see dissemination above). All co-applicants have been asked to identify and report examples of the impact of PPI in the study. A standing agenda item in Co-applicant Meetings reminds everyone to do this. While recognising it can be difficult to attribute decisions to individual input, co-applicants are regularly encouraged to support this attempt to capture this evidence.

*Support for public and patient contributors*

The GPs in EDs project has allocated responsibility for ensuring public and patient involvement in the study to an academic team member. Their role is to facilitate public and patient involvement in the study by supporting public contributors and overseeing any arrangements, information and training required so these individuals have the necessary resources to be actively involved and effectively contribute to the project. Training needs will be reviewed annually. The study has also identified and costed a realistic budget in order to offer honoraria and reimburse expenses incurred by public contributors, in line with best practice.

**Appendix 3: Involvement Episodes in study delivery**

Four accounts of input and impact of public contributors in study management, implementation and scrutiny (see also Table 2)

*Box 3.1: Preparation of Ethics application*

The ethics application and supporting documents are prepared very early in a study. Study researchers proposed to send JH and BH near-final draft documents for comment, including all patient facing documents. JH and BH were told that this was normal practice since other co-applicants (particularly senior academics and clinicians) have limited time. BH and JH explained that this did not meet their understanding of public involvement; they perceived the Ethics process as a core activity for public contributors since all participant information, participant recruitment and data collection tools needed to be included in the application. Rather than review near-final documents by email, they hoped for more active involvement from an earlier stage. The researchers agreed to enable greater involvement in drafting the documentation. The exchange highlighted different understandings of public involvement and led to an agreed way of working. Further meetings were amicable and collaborative. The exchange, soon after the study start, was documented in meeting notes and appeared to help build trust from an early stage by identifying different views and clarifying a shared approach.

*Box 3.2: Patient interviews - response rates*

JH and BH reviewed and amended the patient recruitment information early in the study for the Ethics proposal. During recruitment, site researchers reported low response rates after seeking patients to interview at two study sites. BH and JH suggested reasons why patients may be reluctant to respond to recruitment information. As well as making some further changes to the recruitment letter to encourage a response, they identified ways to enhance response rates:

• using white envelopes instead of brown;

• sending the letter on headed paper from the relevant hospital rather than the university (which was in Wales while study sites were across England);

• giving patients the added option of registering interest via a text instead of posting back the completed consent form

• offering a voucher to each individual who completed an interview

These changes were all adopted. Recruitment levels increased by 2% [38].

*Box 3.3: Qualitative analysis*

JH and BH read a sample of interview transcripts (patients, hospital managers and clinical leads) and discussed themes and their responses, with the site researchers. They also commented on presentation of qualitative data. Their feedback was as follows:

• They believed patient respondents were not typical of all ED attendees and patient behaviours, but a self-selecting group who were more likely to feel their decision to attend ED was justified in some way rather than perceived to be wrong.

• They confirmed that models of GPs in EDs are hugely complex with much variation to reflect local context.

• They felt patient interview results added value to understanding other data. The research team agreed to embed patient interview results with quotations into the other data to ‘add flavour’ and illustrate theories.

• They identified research themes. These were in line with those identified by researchers, who said they were reassured by this confirmation.

• JH and BH reported benefits to themselves from reviewing interview data. They said the insight into data quality, patient experience and complexity of the models helped them undertake their role in the study. Looking at the actual patient transcripts gave them more of a ‘feel’ for the research and made it come alive, they said.

• The researchers and public contributors agreed to collaborate more closely at theory-building stage when JH and BH would check that theories accurately reflected data

*Box 3.4: Dissemination planning*

Public use of emergency departments has a high media profile. To help the research team plan the dissemination strategy, JH offered to seek wider patient views on how to manage study findings. She presented to the SUPER public/patient group [43] and facilitated discussion about messages, timings and media. She collected the following views and reported these to the research team:

• Strong patient interest in the topic

• Report at study end, not interim, to ensure clear message about confirmed results

• Use media interest in stories about EDs to maintain profile and links with the media

• Consider multi-lingual reporting

• Avoid negative language such as ‘inappropriate patients’ or ‘inappropriate attendance’ when describing ED patients

• Match message and media to audience and seek out influencers

JH and BH reviewed the Dissemination and Publication Plan. They recommended a third section on Engagement and provided content for this. They contacted the Communications section of the Public Involvement and Engagement Support Centre at Health and Care Research Wales (https://healthandcareresearchwales.org/index.php/) and agreed joint working on public dissemination.

They co-authored papers, reports and presentations. They advised on terms to describe a patient’s journey through an Emergency Department. These terms (‘streaming’ or ‘redirection’ rather than ‘navigation’; ‘primary care pathway’ rather than ‘models’) were adopted by the research team.

**Appendix 4: Involvement episodes in public involvement processes**

Four accounts of input and impact of public contributors in developing and facilitating public involvement in the study **(see also Table 3)**

*Box 4.1: Public contributors prepared and annually reviewed a PPI plan*

The PPI team included the two public contributors (JH and BH) and PPI lead (BAE). Early in the study, they met to discuss how public involvement would be undertaken in the study. They drafted a document which confirmed the aim of undertaking public involvement and how this would be achieved. This document was reviewed and updated annually to record roles undertaken and new ones identified. Over the study period, their role was widened to include: research tasks such as qualitative data analysis and dissemination of results; public involvement tasks such as auditing public involvement against the UK standards for public involvement [10] and reviewing training needs. The document also recorded that the PPI role was coproduced by the PPI team in response to how the research study was implemented and opportunities which were identified during interaction of the research team members. The annual meeting provided an opportunity for reflection and discussion through the formal structure of the role document.

*Box 4.2: Public contributors’ role in enabling patients to contribute at Stakeholder Events*

Two stakeholder events were held during the study to gain input from clinicians, patients, managers, commissioners, policymakers and academics at key stages of data collection and interpretation. JH and BH had responsibility for supporting patients to participate. They devised a recruitment strategy, recruitment documents and follow-up/thankyou correspondence. They prepared briefing materials. They were involved in planning the meetings to ensure format enabled patient input including: accessible venues; timings to allow for travel and health limitations; the offer of financial support to attendees. They supported patient attendees at the event, co-facilitated discussions and also co-presented at the second event.

Delays to the study timetable impacted on progress of results. This meant that the purpose of the second stakeholder event, previously intended as an occasion for dissemination and action-planning, needed updating. Public contributors were actively involved in defining the revised aim and scope of the event. They expressed concerns that it should make a genuine contribution to the research and patient attendees should have meaningful input. In an email to the Chief Investigator (CI), they queried whether it was good use of time or money to invite patients ‘without them having a clear role and opportunity for input into the study’ and highlighted a possible ‘risk to maintaining the engagement of the public contributors if they are invited to a conference where the reasons for them being there are unclear.’ The CI and research team acknowledged these concerns and worked with JH and BH to propose an agenda which included three discussion sessions allowing review and input to different aspects of the study. JH and BH co-presented a session and facilitated other round-table activities. They also advised how to plan table membership by having at least one patient attendee among other mixed stakeholders.

*Box 4.3: Auditing and improving public involvement in the study*

JH and BH proposed to audit public involvement in the study, using the newly published UK Standards for Public Involvement [10]. Research team members supported this activity. JH facilitated a meeting with core researchers and the Chief Investigator. They reviewed each standard and indicator by identifying evidence of how far the project met each one. They then identified opportunities to improve practice where evidence was weak or non-existent. The completed audit included a list of tasks to improve the quality of public involvement. Against each, a responsible person was nominated. A year later, the list was reviewed by JH. She reported to the research team that all items had been progressed. For example, Involvement Standard 1 concerned Inclusive Opportunities, measured by an indicator to show how widely involvement opportunities are publicised. The team felt better use of social media could help reach a more diverse range of patients, when recruiting people to attend the second Stakeholder Event. This was undertaken when planning the event. Other areas for improvement included: improving research team communication; undertaking a review of PPI training needs; improving the involvement of JH and BH in dissemination activities.

A copy of the audit document is available at Appendix 5.

*Box 4.4: Role of the public contributor members of the Study Steering Committee*

The Study Steering Committee (SSC), which included two public members, received reports of public involvement in the study alongside other information provided for review. They praised the quality of public involvement in the whole study and recommended that data about its impact should be recorded and reported. They also requested that public involvement across the study, including the SSC, be reported in study outputs. After reviewing public involvement processes across the study, they recommended that public involvement should be owned by the whole study team. As evidence of this, all documents concerning public involvement should be jointly authored and agreed. This recommendation aligned with the team’s commitment to equal status among co-applicants and is evidenced by joint authorship of this and other paper.

**Appendix 5: Audit of public involvement**


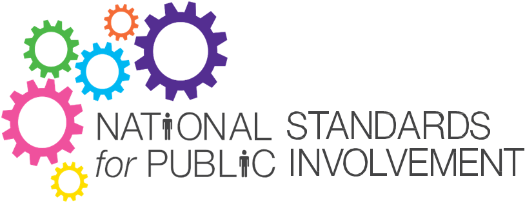

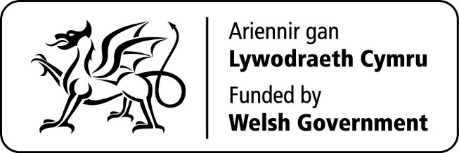

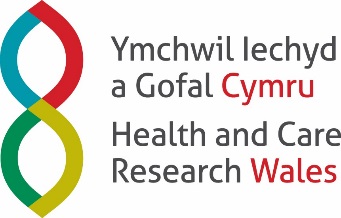


**National Standards for Public Involvement Audit tool template.**

**Actions Required from GPs in EDs audit carried out 7^th^ August 2018**

| **Standard number** | **Indicator** | **Evidence** | **Does the evidence meet the standard?** | | **Actions required**  **Actions achieved as at June 5^th^ 2019 in red below** | **Responsibility** | **Timescale** |
| --- | --- | --- | --- | --- | --- | --- | --- |
|  |  |  | Yes | No |  |  |  |
| **Standard 1: Inclusive Opportunities** | 1.3 We make information about opportunities for public involvement in research available, using different methods so that we reach relevant and interested people | Partial. Advertised in IPN newsletter which goes out to database. Wider range of organisations used for stakeholder recruitment. Some social media used. | √ | √ | Could make more use of social media and look at having contact details for some hard to reach groups such as ethnic minorities, mothers of young children, ED users. Need to look at responses from Stakeholder evaluation questionnaire for new ideas.  Will be looking at how and where we advertise PPI opportunities for Stakeholder meeting. Planning meeting arranged for this event will include discussion on recruiting diverse members of the public. | BN/NP | On-going from Aug 2018 |
| **Standard 1: Inclusive Opportunities** | 1.4 We have fair and transparent processes for involving the public in research | Partial. Fair selection process. Description of opportunity provided to interested people. | √ | √ | Tend to get experienced PPI through IPN. May need to look at more targeted approach to get users of service in this case – advertising in EDs? May be difficult to get certain types of user to engage, eg homeless.  See comment for 1.3 above | BE/NP | For any further recruitment of PPI to project |
| **Standard 2: Working Together** | 2.4 We recognise individual ideas and contributions and uphold decisions together | Partial. Active involvement of PPI is welcomed, PPI contributions are equally valued alongside other meeting partners. PPI contributions are encouraged in meetings and suggestions have been acted upon and recorded. Aware that PPI exists in 3 places in Project – Project Team, Stakeholder Group and Steering Committee. Perhaps Project Team PPI awareness of what Steering Committee PPI are doing could be improved? | √ | √ | Steering Group Minutes. Could these be circulated?  These are now circulated to BH and JH and are proving helpful in painting a fuller picture of all aspects of the project. | BE/NP | All future SC minutes |
| **Standard 3: Support & Learning**  We offer and promote support and learning that builds confidence and skills for public involvement in research. | 3.1 We designate and monitor resources to ensure and support effective public involvement | Partial. Tailored support is provided. Honoraria and travel. Support needs discussed by PPI and BE, NP. Additional support provided for Stakeholder meeting to increase attendance. PPI have access to training provided through HCRW to IP network. | √ | √ | More realistic costings needed perhaps at start of Project to ensure they cover the extent of PPI input required.  Add training review as an agenda item to PPI meetings.  Training review added to agenda for May 2019 PPI meeting. No additional training needs identified at that time.  Costing case study being developed by JH and Barbara Moore to help researchers using new costing guidelines issued 1^st^ April 2019. Costings from GPs in EDs (anonymised) will be used to help develop the case study. | Future Project Managers/CIs  BE | N/A  May 2019 and subsequent meetings |
| **Standard 3: Support & Learning** | 3.4 We develop, deliver and monitor learning opportunities in partnership, for all involved in research | All these apply to work done by the Support Centre for H&CRW so probably not relevant to this individual Project. BE provided a phone induction at the start. | √ | √ | PPI training to be a review item in PPI meetings in future  Done – see 3.1 above | BE/JH/BH | May 2018 and subsequent meetings. |
| **Standard 4: Communications**  We use plain language for timely, two way and targeted communications, as part of involvement plans and activities. | 4.1 We develop and deliver a communications plan for our involvement activities | Communications work well in practice and plain language is used in communications for Co-Applicant Meetings.  PPI role in producing documents for wider dissemination to e.g. the public and participants is acknowledged in the Project Publication and Dissemination Policy and in the Role description for PPI.  Sometimes difficulty in keeping up to speed on developments for PPI when meetings are only every 6 months. | √ | √ | Strengthen PPI input to dissemination plans by adding bullet point to PPI Role Document to state “Ensure via review that wherever possible documents for public and patient contributors are written in plain English”  Sentence added at May 2019 review meeting.  Newly introduced summary of progress produced by NP has solved the problem of updates and this has now been requested by other members of the research team. | BE/JH/BH  NP | Aug 2018  Actioned July 2018 |
| **Standard 5: Impact** | 5.4 We reflect, learn and report the extent to which we have met our intended purpose and predicted outcomes | Partial. Publication on PPI planned for the end of the Project (BE)  Annual meeting minutes include details of impact.  NIHR 6 monthly report reflects on PPI activity and impact.  Annual review of PPI role description involves reflecting on achievement of purpose and amending how to do this if necessary. | √ | √ | Consider formatting the annual PPI review minutes and the role description to show column for date to be achieved in addition to who is responsible for the action.  Done for 2019 PPI review meeting | BE/JH/BH | May 2018 and subsequent meetings. |
| **Standard 6: Governance** | 6.4 We allocate money and other resources for public involvement | Dedicated PPI budget. Paid honoraria and travel. PPI members consulted over PPI support for Stakeholder event. | √ | √ | PPI Costings for any future Project may reviewed in the light of experience gained in this Project in line with comments made under 3.1 | Future Project Managers/CIs | N/A |
